# CM-CPPA: Chaotic Map-Based Conditional Privacy-Preserving Authentication Scheme in 5G-Enabled Vehicular Networks

**DOI:** 10.3390/s22135026

**Published:** 2022-07-03

**Authors:** Mahmood A. Al-Shareeda, Selvakumar Manickam, Badiea Abdulkarem Mohammed, Zeyad Ghaleb Al-Mekhlafi, Amjad Qtaish, Abdullah J. Alzahrani, Gharbi Alshammari, Amer A. Sallam, Khalil Almekhlafi

**Affiliations:** 1 National Advanced IPv6 Centre (NAv6), Universiti Sains Malaysia, Gelugor 11800, Malaysia; m.alshareeda@nav6.usm.my; 2 College of Computer Science and Engineering, University of Ha’il, Ha’il 81481, Saudi Arabia; b.alshaibani@uoh.edu.sa (B.A.M.); ziadgh2003@hotmail.com (Z.G.A.-M.); am.qtaish@uoh.edu.sa (A.Q.); aj.alzahrani@uoh.edu.sa (A.J.A.); gk.alshammari@uoh.edu.sa (G.A.); 3 Engineering and Information Technology College, Taiz University, Taiz 6803, Yemen; amer.sallam@taiz.edu.ye; 4 CBA-Yanbu, Taibah University, Al Madinah 42353, Saudi Arabia; drkhalilalmekhlafi@gmail.com

**Keywords:** 5G-enabled, Chebyshev polynomial, chaotic map, vehicular networks, privacy and security

## Abstract

The security and privacy concerns in vehicular communication are often faced with schemes depending on either elliptic curve (EC) or bilinear pair (BP) cryptographies. However, the operations used by BP and EC are time-consuming and more complicated. None of the previous studies fittingly tackled the efficient performance of signing messages and verifying signatures. Therefore, a chaotic map-based conditional privacy-preserving authentication (CM-CPPA) scheme is proposed to provide communication security in 5G-enabled vehicular networks in this paper. The proposed CM-CPPA scheme employs a Chebyshev polynomial mapping operation and a hash function based on a chaotic map to sign and verify messages. Furthermore, by using the AVISPA simulator for security analysis, the results of the proposed CM-CPPA scheme are good and safe against general attacks. Since EC and BP operations do not employ the proposed CM-CPPA scheme, their performance evaluation in terms of overhead such as computation and communication outperforms other most recent related schemes. Ultimately, the proposed CM-CPPA scheme decreases the overhead of computation of verifying the signatures and signing the messages by 24.2% and 62.52%, respectively. Whilst, the proposed CM-CPPA scheme decreases the overhead of communication of the format tuple by 57.69%.

## 1. Introduction

The vehicular ad hoc networks (VANETs) aim to provide drivers and passengers with smarter, safer, and more entertainment services. In the coming fifth-generation (5G) era, the participating vehicle in the vehicular network is investigated to be installed with an on-board unit (OBU) for vehicle-to-everything (V2X) communication [1,2,3]. Therefore, the vehicle can exchange messages with others and the outside world.

Currently, the number of urban automobiles is quickly increasing, and the demands of drivers are diversifying. Thus, the VANET under the 5G mobile networks can satisfy the characteristics of high bandwidth and wide coverage for the current application environment. It presents a plethora of challenges as well as opportunities for vehicular networks [4,5,6]. Under 5G wireless networks, the characteristics are 20 Gb/s and 100 Mb/s for data transmission and average data transmission rates, respectively [7,8].

Nevertheless, owing to the nature of wireless communication as a public channel, the exchanged messages among vehicles might be modified when adversaries exist. Privacy and security vulnerabilities are critical requirements in a 5G-enabled vehicular network. Therefore, the message must be checked by the receiver to distinguish if it is reliable [9,10,11].

Regardless of privacy and security requirements, effective performance is also critical in V2X communication. For example, when there are 100 nodes within the Covered zone of the vehicle, the receiver demands to verify 334–1500 messages per second. All the data shared by a vehicle can obviously be signed and verified by using cryptography operations to secure communication. However, the most recent conditional privacy-preserving authentication (CPPA) schemes are using operations elliptic curve (EC) or bilinear pair (BP) cryptographies to verify the signatures and sign messages. So, these operations are considered to be more complicated and time-consuming, and these cryptography algorithms are not suitable to be deployed in a 5G-enabled vehicular network.

The motivation behind this paper is to cope with this issue of effective performance during the signing and verifying messages caused by complicated and time-consuming operations. Therefore, this paper will propose a chaotic map-based CPPA scheme for vehicular networks relying on 5G technology. The proposed CM-CPPA scheme employs Chebyshev polynomial mapping operations to secure V2x communication.

To the best of the authors’ knowledge, Chebyshev polynomial mapping operations have not been used for CPPA schemes to date in the literature in 5G-enabled vehicular networks. More precisely, the following is a list of this paper’s key contributions:

- A chaotic map-based CPPA scheme is proposed for vehicular networks relying on 5G technology to achieve security goals in terms of piracy (Identity-Anonymity and Unlinkability) and security (Message Integrity and Authentication and Traceability) requirements. Moreover, our proposal has the ability to resist security attacks, such as modification, impersonation, replay, and man-in-the-middle.
- A scheme that applies chaotic polynomial map to generate/sign message (e.g., signer side) and verify the validity and authenticity of signature (e.g., checker side).
- A scheme that uses the well-known accepted simulator called AVISPA tool under OFMC and CL-AtSe back ends and shows that the scheme is perfectly secure against active and passive attacks.
- A scheme that outperforms most recent related schemes based on EC and BP operations with regard to overhead of computation and communication.

Here is how the remainder of the paper is organized. Section 2 discusses some CPPA schemes. Network structure, security goals and attackers model are explained in Section 3. Section 4 introduces the proposed CM-CPPA scheme. The security proof and performance metric of the proposed CM-CPPA scheme are given in Section 5 and Section 6, respectively. Ultimately, Section 7 provides the conclusion of our proposal.

## 2. Related Work

In this section, some CPPA schemes for vehicular networks are discussed to secure wireless communication instead of public wireless communication.

Raya and Hubaux [12] proposed an approach that is based on anonymous certificates of users instead of the true identities for achieving the exchanged information integrity check. Digital signatures were used in this approach regarding to public key infrastructure (PKI) to introduce the CPPA schemes [12,13,14,15,16,17,18,19,20,21]. In vehicular networks, the PKI-based scheme is a sophisticated choice due to exchange of information that has not yet been determined as reliable and is purposed to be authenticated [10]. However, its limitations are also evident as follows: (i) a tremendous quantity of anonymous certificates are preloaded to each participating registered vehicle for achieving privacy; (ii) a great deal of private/public key pairs are stored in each vehicle, which needs a lot of storage capacities; (iii) all private/public key pairs and anonymous certificates for all participating vehicles are also required to store the trusted authority (TA), which causes burden storage for TA; (IV) disclosing the true identities of misbehaving vehicles in massive registration vehicle lists can also be exhausting.

With the purpose of settling the issues of the PKI-based approach, some researchers proposed an identity (ID) approach in vehicular networks. Instead of preloading a tremendous quantity of pairs (e.g., private/public key) and the relevant certificates, the identification is applied as the vehicle’s key of public, while private keys are issued by a TA utilizing the same identification and then preloaded to participating registered vehicles. The participating registered vehicle signs the message by employing its private key, whereas the verifier checks the integrity and authenticity of the message sent by utilizing the public key of the node. Zhang et al. [22] constructed authentication schemes by supporting the batch verification process to test the validity of multiple messages simultaneously in vehicular communication. Lee and Lai [23] identified two main limitations of the scheme proposed by Zhang et al. [22]. In the first limitation, the node can issue a forged identification to avoid the traceability property. In the second limitation, the impersonation attack is not resisted. To tackle the two limitations, Lee and Lai [23] designed an enhanced ID-based scheme to satisfy the top-level of security in vehicular communication. Jianhong et al. [24] also highlighted two main limitations of the scheme proposed by Lee and Lai [23], in which the non-repudiation property is not satisfied, and the replay attacks are not resisted. To tackle the two limitations, Jianhong et al. [24] provided an enhanced ID-based scheme to satisfy much higher effectiveness. Recently, some authentication schemes have been proposed by [25,26,27,28,29,30] to tackle privacy and security properties for vehicular networks. Furthermore, the ID-based schemes [25,26,27,28,29,30,31] use cryptography operations related to the Bilinear Pair (BP) to verify signatures and sign messages. Nevertheless, the operations of BP are complicated and time-consuming, which creates massive performance overheads with regard to overheads of communication and computation to verify signatures and sign messages. For instance, the participating registered vehicle in the Pournaghi et al. scheme [30] needs one bilinear pair operation and one Point-to-Map hashing for message signing, whereas the verifier needs three bilinear pair operations and one hashing (e.g., Point-to-Map) for message verification.

To overcome the time-consuming operations of ID-based BP approaches, He et al. [32] proposed an ID-based elliptic curve (EC) Cryptography to provide a signature verification process for vehicular communication. Recently, some authentication schemes based on EC have been proposed by [32,33,34,35,36,37,38,39] to tackle privacy and security proprieties for vehicular communication. Nevertheless, the operations of EC are time-consuming with an increase in the number of participating nodes. Therefore, Cui et al. [40] designed an ID-based scheme by adopting a Chaotic Map to sign and verify messages for the VANET. However, Cui et al. [40] used equipment called road-side unit (RSU) for authentication purposes, and RSU is more expensive and often needs thousands of dollars. To remove the used RSU, the most recent existing schemes use a 5G-enabled-vehicular network [41,42,43]. However, these schemes use time-consuming EC operations.

In the PKI approach, the vehicle saves large numbers of key pairs and the relevant certificate into OBU. Then, the vehicle selects randomly one of these parameters to verify the signature and sign the message. Unlike the PKI approach, the vehicle in our proposal only saves the master key of the system and public parameters. Thus, the vehicle uses the master key to generate the signature and compute a pseudonym for each communication in vehicular communication relying on 5G technology. To tackle the above issues arising in the vehicular communication, this paper proposes a chaotic map-based CPPA scheme for vehicular communication relying on 5G technology. Our proposal employs Chebyshev polynomial mapping operations instead of EC and BP operations for the signature verification process.

## 3. Background

This section presents the preliminaries for the proposed CM-CPPA scheme for vehicular communication relying on 5G technology with regards to the network structure, security goals, and attackers model as follows.

### 3.1. Network Structure

**Figure 1:**
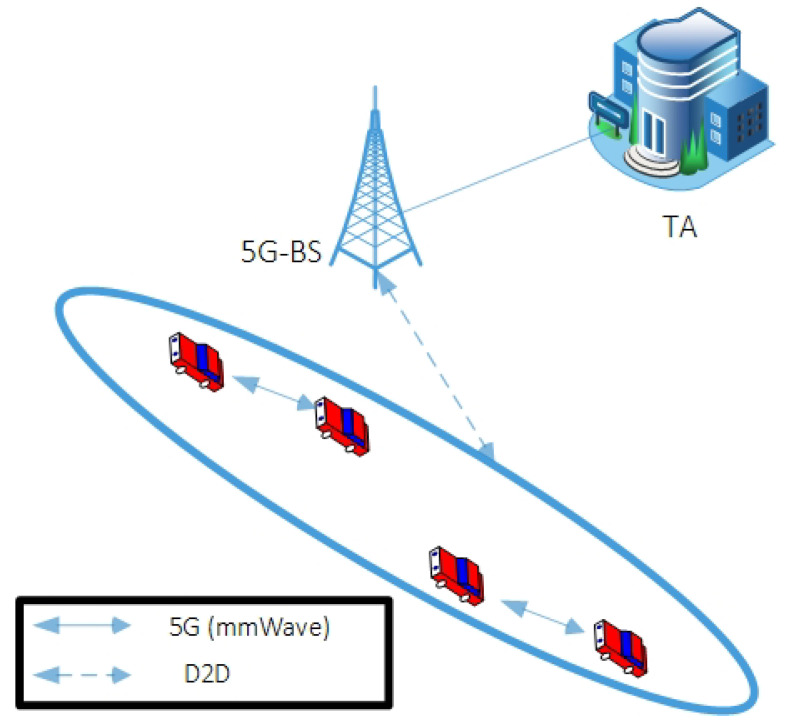
Network Structure of vehicular communication relying on 5G technology [44].

In this section, the explanation of the network structure of our proposal is given, which includes three network components: (1) TA; (2) 5G-BS; and (3) vehicle. The following steps are a brief explanation of these components, as presented in Figure 1.

- TA: Suppose that TA is full trust and has high resources with regard to computation capacity, sufficient storage, and high credibility. In the proposed CM-CPPA scheme, TA is in charge of generating security parameters (chaotic map) and preloading them to participating registered vehicles during the registration process. Furthermore, the TA saves its private key to the vehicle security. If the attacker compromises the TA, the entire system will be insecure. Additionally, the TA has the ability to update the parameters and then preload them into the vehicle to avoid any attack occurring.
- 5G-BS: The 5G-BS is a wireless infrastructure mounted along the sides of roads. Suppose that 5G-BS is hard to be compromised. In the proposed CM-CPPA scheme, 5G-BS only acts as an intermediary transmission medium between the nodes and the TA without any storage or verification.
- Vehicle: Each vehicle has OBU for communicating with each other and sharing traffic information. A tamper-proof device (TPD) is fitted in each OBU to save critical data received from TA.

### 3.2. Security Goals

This subsection describes security goals that will be achieved by the proposed CM-CPPA scheme for vehicular communication relying on 5G technology.

- Privacy (Identity-Anonymity): A true identification of the node must become fully anonymous to all participating registered vehicles.
- Message Integrity and Authentication: The recipient of a message has the ability to validate that the received message has not been changed by an adversary. Furthermore, the recipient of the message can check that it was issued by a participating registered vehicle.
- Traceability: In vehicular communication relying on 5G technology, the TA has the capability to track down the true identity of the sender in case of a dispute.
- Unlinkability: A third party should not be capable of relating several messages or predicting that these were issued by the same sender.
- Resistance to Security Attacks includes: modification, replay, impersonation, and man-in-the-middle attacks.

### 3.3. Attackers Model

Since the message is shared by the vehicle in a public channel, the attacker has the ability to fully control the message by changing, modifying, and replaying it to achieve their own malicious benefit. Security and privacy issues are both serious challenges in vehicular networks. To propose any sophisticated CPPA scheme, the following attacks must be resisted carefully.

- Modification Attack: The message shared by the vehicle can be modified/altered by the attacker to disturb the system.
- Impersonation Attack: The true identification of the node can be impersonated by an attacker to broadcast a forged message in the system.
- Replay Attack: The message shared by vehicles can be replied to at different times to send it again to other vehicles.
- Man-In-The-Middle Attack: The data shared between the recipient and sender can be interrupted by a third party to make damage to the entire system.

## 4. Proposed Scheme

**Figure 2:**
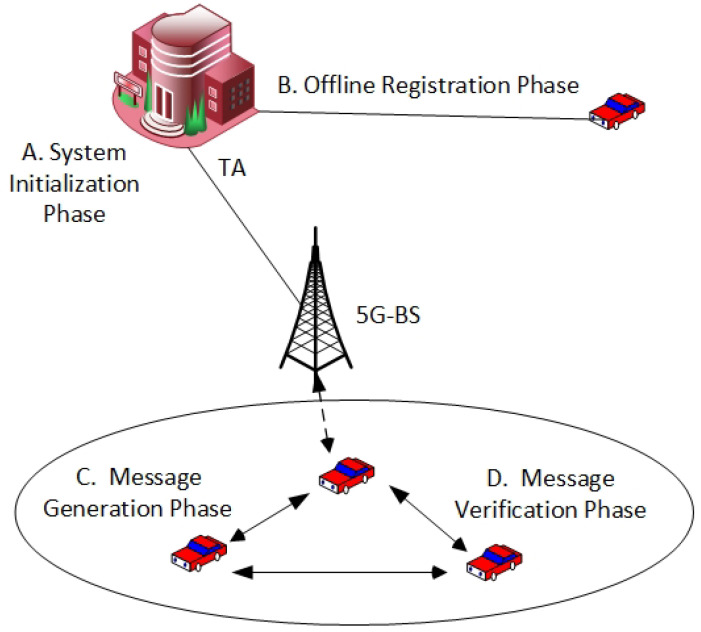
The Proposed CM-CPPA Scheme Phases.

This section presents the proposed CM-CPPA scheme without using the BP or EC operations. As displayed in Figure 2, the four main phases included in the proposed CM-CPPA scheme for vehicular communication relying on 5G technology: the system initialization, offline registration, message generation, and message verification phases. The notations applied in our proposal are defined by the subsequent expression.

- Consider *P* be a large prime number and *n* be an integer.
- Consider *x* be an inconstant tacking value in the interval [−∞,∞]→ [−∞,∞].
- Consider $T_{n}\left(.\right)$ be a chaotic map (e.g., Chebyshev polynomial) of class n, which it is determined according to Equation (1).
  (1) $$cos\left(n*arccos\left(x\right)\right)=T_{n}\left(x\right)$$
- The form of recurrence of the chaotic map $T_{n}:R\to R$ is as Equation (2).
  (2) $$2xT_{n-1}\left(x\right)-T_{n-2}\left(x\right)\left(modP\right),\left(n⩾2\right)\equiv T_{n}\left(x\right)$$
  where $T_{1}\left(x\right)=x$ and $T_{0}\left(x\right)=1$. Additionally, a few instances of the chaotic map are given by Equation (3).
  (3) $$\begin{array}{cc} T_{4}\left(x\right) & =8x^{4}-8x^{2}+1,\\ T_{3}\left(x\right) & =4x^{3}-3x,\\ T_{2}\left(x\right) & =2x^{2}-1 \end{array}$$
- Chaotic feature: If degree 1 < n, it can determine the chaotic map $T_{w}:\left[-\infty ,\infty \right]\to$ [−∞,∞] with a fixed thickness $f^{*}\left(y\right)=1/\left(\pi \sqrt{1-y^{2}}\right)$ function.
- Semi-group feature:
  $$\begin{array}{cc} T_{w}\left(T_{l}\left(x\right)\right) & =cos(wcos^{-1}\left(cos\left(lcos^{-1}\left(x\right)\right)\right))\\ \; & =cos(wlcos^{-1}\left(x\right))\\ \; & =T_{wl}\left(x\right)\\ \; & =T_{l}\left(T_{w}\left(x\right)\right) \end{array}$$
  where *l* and *w* are positive integers and x∈ [−∞,∞].
- Discrete Logarithm (DL) problem: The major function of DL is to detect the unknown number *w* such that $T_{w}\left(x\right)\equiv y$ for two taking items *y* and *x*.
- Diffie–Hellman (DH) problem: The major function of DH is to evaluate the $T_{wl}\left(x\right)$ for three taking items *x*, $T_{l}\left(x\right)$, and $T_{w}\left(x\right)$.
- *h*: The chaotic map-based hash function, where h:${\left[-0,1\right]}^{*}\to$${\left[-0,1\right]}^{l}$.
- $TID_{vi}$: The identity of the vehicle.
- ||: The operation of message concatenation.
- ⊕: The operation of exclusive-OR.
- $M_{i}$: The information-related message.
- $PID_{vi}$: The pseudonym-identification of the vehicle.
- $\sigma_{vi}$: The signature of the safety-related message $M_{i}$.
- $T_{vi}$: The current timestamp.

### 4.1. System Initialization Phase

In this phase, the TA is in charge of issuing system parameters (such as the chaotic map defined on it) before the deployment of 5G-enabled vehicular networks. This phase can be mathematically modeled by the subsequent steps.

- TA randomly picks values $x,k_{s}$ and the large prime *P* based on the chaotic map in the whole system.
- TA randomly picks a value *s* as its private key.
- TA chooses a chaotic map-based hash function *h*, where h: ${\left[-0,1\right]}^{*}\to$ ${\left[-0,1\right]}^{l}$.
- TA sets params= {*x*, $k_{s}$, *P*, *h*} as the public parameters.

### 4.2. Offline Registration Phase

In this phase, the vehicle $V_{i}$ executes the registration process at the system (TA) before leaving the factory. Based on the identity of vehicle $V_{i}$, the system parameters based on a chaotic map are preloaded to participating vehicles in 5G-enabled vehicular networks through a secure channel. The vehicle $V_{i}$ executes the offline registration phase by the subsequent steps.

- The driver submits the true identity of vehicle $TID_{vi}$ to the local TA.
- Once receiving $TID_{vi}$, the TA saves it to the list of vehicle registration.
- TA preloads the public parameters params= {*x*, $k_{s}$, *P*, *h*} to the vehicle $V_{i}$.
- Finally, TA saves its private key *s* to the TPD of vehicle $V_{i}$.

Note that sensitive data are stored in TPD, which will be never disclosed. Therefore, the adversary cannot successfully launch any attack to retrieve the private key of the system in the proposed CM-CPPA scheme. Additionally, our proposal only supports offline registration rather than online registration in order to avoid any insider attack in the system. Once this attack accrued, the forged message broadcasts to all vehicles to disrupt the system. Therefore, before the vehicle leaves the factory, the above steps should be carefully executed.

### 4.3. Message Generation Phase

**Figure 3:**
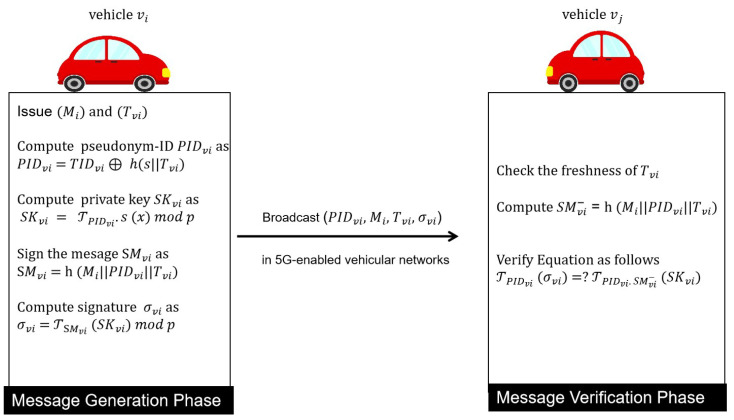
Message Generation and Verification Phases.

In this phase, the TPD of vehicle $V_{i}$ issues a pseudonym-ID $PID_{vi}$ and signature $\sigma_{vi}$ of safety-related message $M_{i}$. After that, vehicle $V_{i}$ broadcasts the pseudonym-ID $PID_{vi}$, the message $M_{i}$, and the signature $\sigma_{vi}$ to neighboring nodes in 5G-enabled vehicular networks. As shown in Figure 3, the subsequent steps are executed by the vehicle $V_{i}$ in this phase.

- $V_{i}$ issues safety-related message $M_{i}$ and the current timestamp $T_{vi}$.
- $V_{i}$ generates pseudonym-ID $PID_{vi}=$ $TID_{vi}⊕h(s||T_{vi})$.
- $V_{i}$ computes the parameter $SK_{vi}=T_{PID_{vi}.s}\left(x\right)modP$.
- $V_{i}$ signs the message $SM_{vi}=h(M_{i}||PID_{vi}\left|\right|T_{vi})$
- $V_{i}$ computes the signature of safety-related message $\sigma_{vi}=$ $T_{SM_{vi}}\left(SK_{vi}\right)modP$.
- Finally, $V_{i}$ broadcasts the message-tuple {$PID_{vi}$, $M_{i}$, $T_{vi}$, $\sigma_{vi}$} to the participating vehicles nearby in 5G-enabled vehicular networks.

### 4.4. Message Verification Phase

In this phase, the verifier $V_{j}$ can authenticate and validate the signature of a safety-related message before accepting the message for further processing. By utilizing the message verification, no adversary has the ability to impersonate a participating registered vehicle and avoid false message transmission. As shown in Figure 3, the message verification is described by the subsequent steps.

- When receiving the message-tuple {$PID_{vi}$, $M_{i}$, $T_{vi}$, $\sigma_{vi}$}, the verifier $V_{j}$ initially checks the freshness of timestamp $T_{vi}$.
- Then, the verifier $V_{j}$ computes $SM_{vi}^{-}=h(M_{i}||PID_{vi}\left|\right|T_{vi})$.
- Finally, the verifier $V_{j}$ uses the message signature $\sigma_{vi}$ of the message-tuple {$PID_{vi}$, $M_{i}$, $T_{vi}$, $\sigma_{vi}$} to check the safety-related message $M_{i}$, where $\sigma_{vi}=T_{SM_{vi}}\left(SK_{vi}\right)modP$. The verifier $V_{j}$ accepts the message if Equation (4) holds. Otherwise, the message is discarded.

(4) $$\begin{array}{cc} T_{PID_{vj}}\left(\sigma_{vi}\right) & \overset{?}{=}T_{PID_{vj}.SM_{vi}^{-}}\left(SK_{vi}\right) \end{array}$$

## 5. Security Analysis

In this section, the formal (AVISPA simulator) security proof and informal security analysis of the proposed CM-CPPA scheme are evaluated in detail in the following two subsections.

### 5.1. AVISPA

In this subsection, formal security verification of the proposed scheme is done utilizing the well-known “automated verification internet security protocol and applications (AVISPA)” simulator tool for ensuring that the proposed scheme is secure against all attacks. In AVISPA, each player performs a specific role since it is a role-based simulator. The specifications of simulation experiment parameters for AVISPA that had been used are explained as follows. The main tool SPAN Version 1.6 is based on Windows 7 Pro operating system (64 bit) that is running by SPAN-Ubuntu10.10-light on Oracle VirtualBox 6.1, Intel (R) Core (TM) 2.90 GHz processor i7-7500U CPU @ 2.70 GHz and 16 GB RAM.

To validate the proposed CM-CPPA scheme utilizing the AVISPA tool, initially, the proposed scheme is coded by using HLPSL. Then, the HLPSL2IF translator translates the HLPSL code to an IF, where IF is a lower-level language compared to HLPSL. Lastly, the IF specification is utilized directly by the back ends for analyzing whether the security goals are fulfilled or not. According to the output, the AVISPA simulator produces the result either in SAFE or UNSAFE mode against man-in-the-middle attacks and replay attacks. In the current version, the AVISPA simulator offers four types of the following back-ends verification tools: SATMC, CL-AtSe, OFMC, and TA4SP.

**Figure 4:**
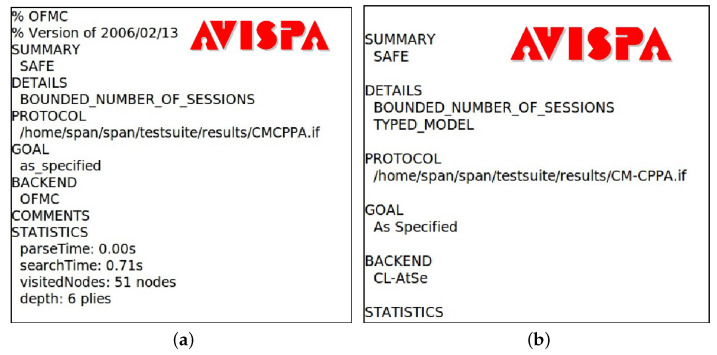
The simulation results under OFMC and CL-AtSe Back Ends. (**a**) OFMC back end; (**b**) CL-AtSe back end.

The authentication and broadcasting phases for the proposed CM-CPPA scheme are coded in HLPSL using two basic roles for an OBU and RSU. This process defines the session, environment, and goal as the compulsory roles. Figure 4 shows the simulation results of the proposed CM-CPPA scheme that resists the man-in-the-middle and replay attacks under the OFMC, and CL-AtSe back ends.

### 5.2. Security Proof

In this section, we provide the security proof of our proposal. The security model is defined as in [40].

**Theorem** **1.** *Consider that the DHP problem based on the hash function is secure and the expanded chaotic mapping is legitimated. Subsequently, according to the DHP problem, the FC-CPPA proposal is the security key negotiation process.*

**Proof.**  Let *A* represent an attacker, apply $q_{k}$ to show the the amount queried by **Send**, apply $q_{s}$ to show the amount queried by **Reveal**, and $q_{e}$ represents the amount of times *A* queried by **Execute**. Build a Challenger *C* to simulate the strong proposal run through a **Send** Oracle query. Depict Game ${\mathrm{Game}}_{i}$: i = 0, 1, 2 *…*, by everlastingly modifying the Oracle answers of adjacent games; it can be presented by the difference in the probability of *A* obtaining the negligible game.

For the end game, the probability of analyzing *A* profitably was only $\frac{1}{2}$. Thus, it is estimated that the probability of a win of the *A* process can be neglected. Considering the event of Repeat indicates that the simulation running instance has picked the $x_{i}$ that has been picked. The probability of the strong event is:

(5) $$Pr\left[Repeat\right]\leq \frac{{\left(q_{k}\right)}^{2}}{2^{k+1}}$$

**Guess:** Once these queries are executed, *A* outputs guess $b^{-}$ of *b*. Once $b^{-}$ = b, then *A* has broken the system security properly. This event is represented by $Succ_{A}$, and $Att_{A}$ has identified the attack feature of any *A* against the scheme, where $Att_{A}\left[2Pr\left[Succ_{A}\right]-1\right]$.

**Game1:***C* answers to *A*’s query according to the running cost operation of the strong process. So, the gaining probability against the *A* is equal to the gaining probability of the *A* attacking the real process. Thereby, we can conclude:

(6) $$Pr\left[E_{0}\right]=Pr\left[Succ_{A}\right]$$

**Game2:** In game **Game2**, all Oracle will rely on **Game1**. Once Forge or Repeat events come, *C* finishes the game. So, we can conclude:

(7) $$Pr\left[E_{1}\right]-Pr\left[E_{0}\right]\leq Pr\left[Repeat\right]+Pr\left[Forge\right]$$

**Game3**: In game **Game3**, for the Send($\sum_{v}^{i},M_{2}$) query, *C* first verifies when the running instance is Corrupt and properly passes it to $\sum_{v}^{i}$, and *C* picks randomly the signature to a random value $SM_{vi}$. Since the signature is a uniformly distributed random value, we can conclude:

(8) $$Pr\left[E_{2}\right]=\frac{1}{2}$$

By combining Equations (4)–(7), we can conclude:

(9) $$Att_{A}=\left|2\cdot Pr\left[Succ_{A}\right]1\right|\leq \frac{{\left(q_{k}\right)}^{2}}{2^{k+1}}+2Att_{dlp}+2\cdot q_{k}Att_{DHP}$$

In the end, according to these games, the probability of an attacker breaking the security of our proposal is negligible. □

### 5.3. Informal Security

The proposed CM-CPPA scheme will achieve the privacy and security requirements as well as it will be proven that the proposed scheme is resistant to various attacks by the following steps.

- Privacy (identity-anonymity): In this work, the participating registered vehicle $V_{i}$ transmits the message-tuple {$PID_{vi}$, $M_{i}$, $T_{vi}$, $\sigma_{vi}$} to nearby others. The true identity of vehicle $TID_{vi}$ is hidden to the message-tuple by $PID_{vi}$=$TID_{vi}⊕h(s||T_{vi})$, where *s* is the private key of the system and $T_{vi}$ is the current timestamp. During the offline registration phase (Section 4.2), once the true identity of the vehicle $TID_{vi}$ is submitted by the driver, the TA saves it to the vehicle registration list. Moreover, when the message-tuple {$PID_{vi}$, $M_{i}$, $T_{vi}$, $\sigma_{vi}$} is captured by a third party, there is no information about the identity of vehicle $TID_{vi}$. Therefore, the identity of the vehicle $TID_{vi}$ is the only known by the participating registered vehicle $V_{i}$ and the TA. So, this work achieves the privacy (identity-anonymity) requirement in 5G-enabled vehicular networks.
- Message Authentication and Integrity: In the proposed CM-CPPA scheme, the message-tuple {$PID_{vi}$, $M_{i}$, $T_{vi}$, $\sigma_{vi}$} is included as a sophisticated signature $\sigma_{vi}=T_{SM_{vi}}\left(SK_{vi}\right)modP$, where $SM_{vi}=h(M_{i}||PID_{vi}\left|\right|T_{vi})$, $SK_{vi}=T_{PID_{vi}.s}\left(x\right)modP$ and $PID_{vi}$=$TID_{vi}$ $⊕h(s||T_{vi})$ (Section 4.3). Based on the complexity of DLP associated with the chaotic map, the malicious vehicle ($V_{m}$) cannot find/retrieve the private key of the system *s* such that $T_{s}\left(x\right)\equiv y$ for two given parts *x* and *y*. Therefore, the verifier $V_{j}$ can confirm the authenticity and integrity of message-tuple {$PID_{vi}$, $M_{i}$, $T_{vi}$, $\sigma_{vi}$} sent from vehicle $V_{i}$ by checking whether equation $T_{PID_{vj}}\left(\sigma_{vi}\right)\overset{?}{=}T_{PID_{vj}.SM_{vi}^{-}}\left(SK_{vi}\right)$ holds (Section 4.4). For instance, after capturing the message-tuple {$PID_{vi}$, $M_{i}$, $T_{vi}$, $\sigma_{vi}$} sent from $V_{i}$, a malicious vehicle ($V_{m}$) modifies/changes the safety-related message $M_{i}$ to a bogus message $M_{f}^{-}$ and then sends the altered false message-tuple {$PID_{vi}$, $M_{f}^{-}$, $T_{vi}$, $\sigma_{vi}$} into 5G-enabled vehicular networks. The verifier $V_{j}$ confirms the authenticity and integrity of the altered false message-tuple {$PID_{vi}$, $M_{f}^{-}$, $T_{vi}$, $\sigma_{vi}$} by checking whether Equation (4) holds. If it holds, then the proposed CM-CPPA scheme achieves the message authenticity and integrity requirements.
- Traceability: In the proposed CM-CPPA scheme, the TA is responsible to register the vehicle based on an unique true identity of vehicle $TID_{vi}$ in the offline registration phase (Section 4.2). Furthermore, there is no publicly available information with respect to the true identity of vehicle $TID_{vi}$ on the message-tuple {$PID_{vi}$, $M_{i}$, $T_{vi}$, $\sigma_{vi}$}, where $PID_{vi}$=$TID_{vi}⊕h(s||T_{vi})$. Therefore, the TA has the capability to retrieve the true identity of vehicle $TID_{vi}$ from a pseudonym-ID $PID_{vi}$=$TID_{vi}⊕h(s||T_{vi})$ included on the message-tuple {$PID_{vi}$, $M_{i}$, $T_{vi}$, $\sigma_{vi}$} by executing $TID_{vi}=PID_{vi}⊕h(s||T_{vi})$ since TA has its private key *s* and obtained timestamp $T_{vi}$. Thus, this work achieves the traceability requirement.
- Unlinkability: In the proposed CM-CPPA scheme, the vehicle $V_{i}$ generates a new pseudonym-ID $PID_{vi}^{new}$=$TID_{vi}⊕h(s||T_{vi}^{new})$ to each message generation process, where $T_{vi}^{new}$ is the new current timestamp. According to the chaotic map-based hash function h(.), the result will change from the various initial input. Therefore, the adversary cannot link two or more message-tuples {$PID_{vi}^{new1}$, $M_{i}^{new1}$, $T_{vi}^{new1}$, $\sigma_{vi}^{new1}$}, {$PID_{vi}^{new2}$, $M_{i}^{new2}$, $T_{vi}^{new2}$, $\sigma_{vi}^{new2}$} sent from the same sender (vehicle $V_{i}$). Thus, the proposed CM-CPPA scheme achieves the unlinkability requirement.
- Resistance to modification attack: The main malicious task of the modification attack is to launch a modified safety-related message $M_{i}$ to falsify the message $M_{f}^{-}$ and then broadcast the altered false message-tuple {$PID_{vi}$, $M_{f}^{-}$, $T_{vi}$, $\sigma_{vi}$} in 5G-enabled vehicular networks. As a result, the entire the system will be insecure. To resist the modification attack, the verifier $V_{j}$ in the proposed CM-CPPA scheme can distinguish any alteration in {$PID_{vi}$, $M_{f}^{-}$, $T_{vi}$, $\sigma_{vi}$} by checking whether equation $T_{PID_{vj}}\left(\sigma_{vi}\right)\overset{?}{=}T_{PID_{vj}.SM_{vi}^{-}}\left(SK_{vi}\right)$ holds. If it holds, then the receiver $V_{j}$ accepts the message $M_{i}$; otherwise, it will be discarded. Hence, the proposed CM-CPPA scheme successfully withstands the modification attack.
- Resistance to impersonation attack: The main malicious task of an impersonation attack is to impersonate a forged message signature $\sigma_{vi}^{f}$ as participating registered vehicle and then broadcast the forged message-tuple {$PID_{vi}$, $M_{i}$, $T_{vi}$, $\sigma_{vi}^{f}$} in vehicular communication relying on 5G technology. Furthermore, the attacker must initially obtain/retrieve the identity of vehicle $TID_{vi}$ and the private key of the system *s* to impersonate a legitimate vehicle. Thus, in this work, vehicle $V_{i}$ hides its $TID_{vi}$ into a pseudonym-ID $PID_{vi}$=$TID_{vi}⊕h(s||T_{vi})$ and the private key of the system *s* is stored in the TPD of the participating registered vehicle $V_{i}$. According to DLP of the chaotic map, the adversary cannot find the private key of the system. Thus, the proposed CM-CPPA scheme successfully withstands the impersonation attack.
- Resistance to replay attack: The main malicious task of replay attack is to replay the previously generated message-tuple {$PID_{vi}$, $M_{i}$, $T_{vi}$, $\sigma_{vi}$} sent from a participating registered vehicle $V_{i}$ to disturb the 5G-enabled vehicular networks. However, in this work, a timestamp $T_{vi}$ is contained in the message-tuple {$PID_{vi}$, $M_{i}$, $T_{vi}$, $\sigma_{vi}$}, where $\sigma_{vi}=T_{SM_{vi}}\left(SK_{vi}\right)modP$ and $SM_{vi}=h(M_{i}||PID_{vi}\left|\right|T_{vi})$. The freshness timestamp is issued by the vehicle $V_{i}$ during the message generation process. Once receiving the message-tuple {$PID_{vi}$, $M_{i}$, $T_{vi}$, $\sigma_{vi}$}, the verifier $V_{j}$ initially checks the freshness of the timestamp to continue the verification process. Otherwise, the message will be discarded immediately. Thus, the proposed CM-CPPA scheme successfully withstands the replay attack.
- Resistance to Man-In-The-Middle (MITM) Attack: The main malicious task of MITM attack is to intercept the participating registered sender $V_{i}$ and receiver $V_{j}$. However, in the proposed CM-CPPA scheme, mutual authentication among nodes is executed. Furthermore, by using the AVISPA tool (Section 5.1), this work is secure against high control attackers (Dolev–Yao model) over the system. Thus, the proposed CM-CPPA scheme successfully resists the MITM attack in 5G-enabled vehicular networks.

## 6. Performance Evaluation

In this section, we analyze and compare the performance of the proposed CM-CPPA scheme with the three most recent schemes based on BP and EC operations.

### 6.1. Computation Overhead Analysis and Comparison

In this section, we analyze CPPA schemes by comparing the overhead of computation of our proposal and the existing schemes for 5G-enabled vehicular networks. For a fair comparison, some cryptography operations about the running cost are as follows.

- $T_{pair-bp}$: the running cost of the BP operation $\overset{-}{e}$ (S, T), where $\overset{-}{S}$, $\overset{-}{T}$∈$G_{1}$.
- $T_{ptm}$: the running cost of a Point-to-Map hashing operation for the BP in $G_{1}$.
- $T_{mul-ec}$: the running cost of operation with regards to a scale multiplication x.P for the EC, where $x\in Z_{q}^{*}$ and P∈G.
- $T_{chev}$: the running cost of Chebyshev’s polynomial mapping operation.
- $T_{h-chec}$: the running cost of the chaotic map hashing operations.

In this paper, negligible operations such as XOR and concatenation are not considered, and only the most significant operations are included. The hardware specification of the Windows 10 operating system contains an Intel I5-3570 processor with 16.0 gigabytes of memory and a 3.40 GHz clock frequency. The running cost of the above cryptographic operations is tabulated in Table 1.

The existing schemes applied ECC and bilinear pairing operations to generate/sign messages and verify the validity and authenticity of the signature. These operations are considered time-consuming and complicated operations. According to Table 1, we can observe that the running costs of bilinear pair operation and scalar multiplication operation are 1.537 ms and 0.715 ms, respectively. In contrast, the running cost of the chaotic polynomial map for our work is 0.341 ms to generate/sign messages and verify the validity and authenticity of signatures for 5G-enabled vehicular networks. The percentage improvement for the running cost of chaotic polynomial map over bilinear pair and elliptic curve are about $\frac{1.537-0.341}{1.537}\approx$ 77.81% and $\frac{0.715-0.341}{0.715}\approx$ 52.31%, respectively.

This paper shows the detailed analysis of the Pournaghi et al. [30], Zhang et al. [41], Cui et al. [42] schemes and the proposed CM-CPPA scheme. The comparisons of computation Overhead for message signing and message verification steps are tabulated in Table 2.

For the message signing step of the Pournaghi et al. scheme [30], the signer requires to run one the BP operation and one Point-to-Map hashing operation for the BP. Hence, the running cost of the step is $1T_{pair-bp}+1T_{ptm}\approx 2.474$ ms. For the message verification step of the Pournaghi et al. scheme [30], the verifier requires to execute three BP operations and one Point-to-Map hashing operation for the BP. Hence, the running cost of the step is $3T_{pair-bp}+1T_{ptm}\approx 5.548$ ms.

For the message signing step of the Zhang et al. scheme [41], the vehicle requires to run three scale multiplication operations for the EC. Hence, the running cost of the step is $3T_{mul-ec}\approx 2.145$ ms. For the message verification step of the Zhang et al. scheme [41], the verifier requires to run two-scale multiplication operations for the EC. Hence, the running cost of the step is $2T_{mul-ec}\approx 1.43$ ms.

For both the message signing step and message verification step of the Cui et al. scheme [42], the vehicle and verifier require to run three scale multiplication operations for the EC. Hence, the running cost of the message signing step and message verification step are $3T_{mul-ec}\approx 2.145$ ms and $3T_{mul-ec}\approx 2.145$ ms, respectively.

For the message signing step of the proposed CM-CPPA scheme, the signer requires to run two Chebyshev’s polynomial mapping operations and two chaotic map hashing operations. Hence, the running cost of the step is $2T_{chev}+2T_{h-chec}\approx 0.804$ ms. For the message verification step of the proposed CM-CPPA scheme, the checker requires to run three Chebyshev’s polynomial mapping operations and one chaotic map hashing operation. Hence, the running cost of the step is $3T_{chev}+1T_{h-chec}\approx 1.084$ ms.

**Figure 5:**
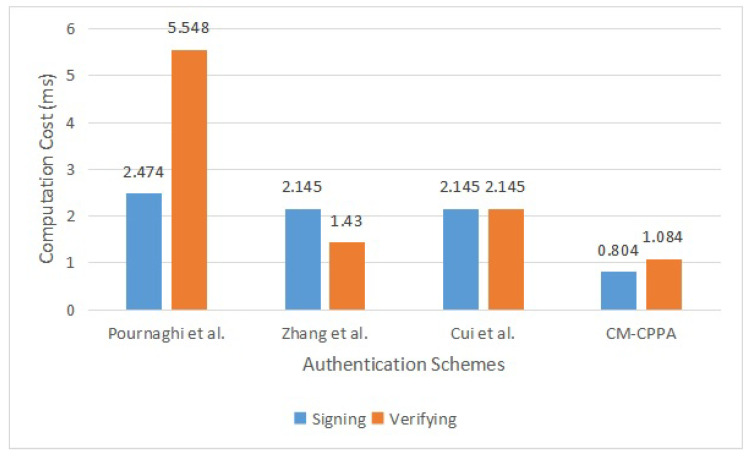
Computation Overhead for Message Signing and Message Verification Steps.

From Table 2, it can be concluded that the proposed CM-CPPA has lower computation overhead compared to other CPPA schemes at the message signing step since the proposed CM-CPPA scheme needs only 0.804 ms while the other existing schemes of Pournaghi et al. [30], Zhang et al. [41], Cui et al. [42] need 2.474 ms, 2.145 ms, 2.145 ms, respectively. Moreover, the proposed CM-CPPA scheme has the lower computation overhead compared to other CPPA schemes at the message verification step because the proposed CM-CPPA scheme needs only 1.084 ms while the other existing schemes of Pournaghi et al. [30], Zhang et al. [41], Cui et al. [42] need 5.548 ms, 1.43 ms, 2.145 ms, respectively. Figure 5 presents the computation overhead for message signing and message verification steps, which illustrates that the proposed CM-CPPA has a lower overhead compared to the CPPA schemes.

The percentage improvement with the message signing and message verification steps of the proposed CM-CPPA scheme over the Pournaghi et al. scheme [30], Zhang et al. scheme [41] and Cui et al. scheme [42] for the total running cost. Table 3 illustrates the improvement of the proposed CM-CPPA over three CPPA schemes.

### 6.2. Communication Overhead Analysis and Comparison

In this section, we analyze CPPA schemes by comparing the overhead of communication for the Pournaghi et al. [30], Zhang et al. [41], Cui et al. [42] schemes and the proposed CM-CPPA scheme in vehicular communication relying on 5G technology. More precisely, in an open channel environment, the communication cost is generated between a message-tuple being broadcasted from a vehicle to others. Our work assumes that the produce of a timestamp is 32 bits, the produce of the hash function is 160 bits, the produce of EC point is (160 + 160) = 320 bits, and the produce of BP point $P=(P_{x},P_{y})$ is (512 + 512) = 1024 bits. The comparison of communication overhead is tabulated in Table 4.

In the Pournaghi et al. scheme [30], the vehicle broadcasts the pseudonym-ID and message signature {$pID_{i}$, $\sigma_{i}$, $M_{i}$, $ID_{RSU_{j}}$} to the verifier, where $pID_{i}=\left\{pID_{i}^{1},pID_{i}^{2}\right\}$, $pID_{i}^{1}$ is the BP point and {$pID_{i}^{2}$, $ID_{RSU_{j}}$, $\sigma_{i}$} are hash functions. Hence, the communication cost of the Pournaghi et al. scheme [30] is 1024 ∗ 2 + 160 ∗ 3 = 2528 bits.

In the scheme of Zhang et al. [41], the signer sends the pseudonym-ID and message signature {$PID_{j}$, $M_{j}$, $Y_{j}$, $S_{j}$, $T_{j}$} to the verifier, where $PID_{j}=\left\{PID_{j,1},PID_{j,2}\right\}$, $PID_{j,1}$ is the EC point, {$PID_{j,2}$, $Y_{j}$, $S_{j}$} are hashing functions and $T_{j}$ is the timestamp. Hence, the communication cost of the Zhang et al. scheme [41] is 320 + 160 ∗ 3 + 32 = 832 bits.

In the scheme of Cui et al. [42], the signer sends the pseudonym-ID and message signature {$PID_{j}$, $DT_{ij}$, $\sigma_{j}$, $D_{j}$, $T_{j}$} to the verifier, where $PID_{j}=\left\{PID_{j,1},PID_{j,2}\right\}$, $PID_{j,1}$ and $D_{j}$ is the EC point, {$PID_{j,2}$, $DT_{ij}$, $\sigma_{j}$} are hashing functions, and $T_{j}$ is the timestamp. Hence, the communication cost of the Cui et al. scheme [42] is 320 ∗ 2 + 160 ∗ 3 + 32 = 1152 bits.

In the scheme of proposed CM-CPPA, the signer sends the pseudonym-ID and message signature {$PID_{vi}$, $M_{i}$, $T_{vi}$, $\sigma_{vi}$} to the verifier, where $PID_{vi}$ and $\sigma_{vi}$ are a hash function and $T_{vi}$ is the timestamp. Hence, the communication cost of the proposed CM-CPPA scheme is 160 *2 + 32 = 352 bits.

**Figure 6:**
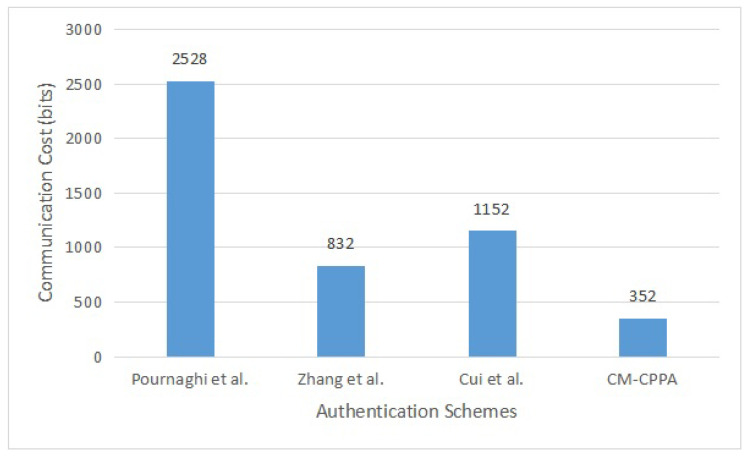
Communication Overhead for Message Tuple.

Figure 6 presents the communication overhead for the message tuple, which illustrates that the proposed CM-CPPA scheme has a lower overhead compared to the recent schemes based on BP and EC operations.

The percentage improvements with the communication overhead of the proposed CM-CPPA scheme over Pournaghi et al. [30], Zhang et al. [41], and Cui et al. [42] for the total message tuple cost are about $\frac{2528-352}{2528}\approx$ 86.08% $\frac{832-352}{832}\approx$ 57.69%, and $\frac{1152-352}{1152}\approx$ 69.44%, respectively.

## 7. Conclusions

This paper proposed an efficient CM-CPPA scheme that employs a Chebyshev polynomial mapping operation and a chaotic map-based hash function to secure communication in vehicular communication relying on 5G technology. A formal Security analysis is applied in our proposal for AVISPA, and the results presented in our work are safe against common attacks. On the other hand, an informal security analysis of the proposed CM-CPPA scheme shows that it achieves the security goals with regard to privacy and security properties. Ultimately, owing to the fact that the proposed CM-CPPA scheme does not employ BP and EC operations, the performance overhead of the proposed CM-CPPA scheme has better efficiency with regards to overheads of communication and computation compared to most recent existing approaches.

In future work, our proposal based on a chaotic map will be satisfied with batch verification to verify several messages simultaneously in vehicular communication relying on 5G technology. Additionally, we will investigate some related schemes that have strong assumptions to propose realistic TPD instead of ideal TPD in our work.

## Figures and Tables

**Table 1:** The Running Cost of Particular Cryptography Operations.

Cryptography Operation	Running Cost (ms)
$T_{pair-bp}$	1.537
$T_{ptm}$	0.937
$T_{mul-ec}$	0.715
$T_{chev}$	0.341
$T_{h-chec}$	0.061

**Table 2:** Comparison of Computation Overhead.

Schemes	Message Signing	Message Verification	Operations Based
Pournaghi et al. [30]	$1T_{pair-bp}+1T_{ptm}\approx 2.474$ ms	$3T_{pair-bp}+1T_{ptm}\approx 5.548$ ms	Bilnear Pair
Zhang et al. [41]	$3T_{mul-ec}\approx 2.145$ ms	$2T_{mul-ec}\approx 1.43$ ms	Elliptic Curve
Cui et al. [42]	$3T_{mul-ec}\approx 2.145$ m	$3T_{mul-ec}\approx 2.145$ m	Elliptic Curve
CM-CPPA	$2T_{chev}+2T_{h-chec}\approx 0.804$ ms	$3T_{chev}+1T_{h-chec}\approx 1.084$ ms	Chaotic Map

**Table 3:** Improvement of the Proposed CM-CPPA Over Three CPPA schemes.

Schemes	Message Signing	Message Verification
Pournaghi et al. [30]	$\frac{2.474-0.804}{2.474}\approx$ 67.51%	$\frac{5.548-1.084}{5.548}\approx$ 80.46%
Zhang et al. [41]	$\frac{2.145-0.804}{2.145}\approx$ 62.52%	$\frac{1.43-1.084}{1.43}\approx$ 24.2%
Cui et al. [42]	$\frac{2.145-0.804}{2.145}\approx$ 62.52%	$\frac{2.145-1.084}{2.145}\approx$ 49.46%

**Table 4:** Comparison of Communication Overhead.

Schemes	Message Tuple	Size of Tuple	Operations Based
Pournaghi et al. [30]	{$pID_{i}$, $\sigma_{i}$, $M_{i}$, $ID_{RSU_{j}}$}	2528 bits	Bilnear Pair
Zhang et al. [41]	{$PID_{j}$, $M_{j}$, $Y_{j}$, $S_{j}$, $T_{j}$}	832 bits	Elliptic Curve
Cui et al. [42]	{$PID_{j}$, $DT_{ij}$, $\sigma_{j}$, $D_{j}$, $T_{j}$}	1152 bits	Elliptic Curve
CM-CPPA	{$PID_{vi}$, $M_{i}$, $T_{vi}$, $\sigma_{vi}$}	352 bits	Chaotic Map

## Data Availability

Data sharing not applicable.

## References

[1] Lai C., Lu R., Zheng D., Shen X. (2020). Security and privacy challenges in 5G-enabled vehicular networks. IEEE Netw..

[2] Cheng X., Zhang R., Yang L. (2018). Wireless toward the era of intelligent vehicles. IEEE Internet Things J..

[3] Al-Shareeda M.A., Anbar M., Manickam S., Hasbullah I.H. (2022). A Secure Pseudonym-Based Conditional Privacy-Preservation Authentication Scheme in Vehicular Ad Hoc Networks. Sensors.

[4] Al-Shareeda M.A., Anbar M., Manickam S., Khalil A., Hasbullah I.H. (2021). Security and Privacy Schemes in Vehicular Ad-Hoc Network with Identity-Based Cryptography Approach: A Survey. IEEE Access.

[5] Alazzawi M.A., Al-behadili H.A., Srayyih Almalki M.N., Challoob A.L., Al-shareeda M.A. (2020). ID-PPA: Robust identity-based privacy-preserving authentication scheme for a vehicular ad-hoc network. Proceedings of the International Conference on Advances in Cyber Security.

[6] Cheng X., Zhang R., Chen S., Li J., Yang L., Zhang H. (2018). 5G enabled vehicular communications and networking. China Commun..

[7] Al-Shareeda M.A., Manickam S., Mohammed B.A., Al-Mekhlafi Z.G., Qtaish A., Alzahrani A.J., Alshammari G., Sallam A.A., Almekhlafi K. (2022). Chebyshev Polynomial-Based Scheme for Resisting Side-Channel Attacks in 5G-Enabled Vehicular Networks. Appl. Sci..

[8] Prasad K.S.V., Hossain E., Bhargava V.K. (2017). Energy efficiency in massive MIMO-based 5G networks: Opportunities and challenges. IEEE Wirel. Commun..

[9] Al-Shareeda M.A., Anbar M., Hasbullah I.H., Manickam S. (2020). Survey of authentication and privacy schemes in vehicular ad hoc networks. IEEE Sens. J..

[10] Cui J., Wei L., Zhang J., Xu Y., Zhong H. (2018). An efficient message-authentication scheme based on edge computing for vehicular ad hoc networks. IEEE Trans. Intell. Transp. Syst..

[11] Hamdi M.M., Mustafa A.S., Mahd H.F., Abood M.S., Kumar C., Al-shareeda M.A. Performance analysis of QoS in MANET based on IEEE 802.11 b. Proceedings of the 2020 IEEE International Conference for Innovation in Technology (INOCON).

[12] Raya M., Hubaux J.P. (2007). Securing vehicular ad hoc networks. J. Comput. Secur..

[13] Cincilla P., Hicham O., Charles B. Vehicular PKI scalability-consistency trade-offs in large scale distributed scenarios. Proceedings of the 2016 IEEE Vehicular Networking Conference (VNC).

[14] Huang D., Misra S., Verma M., Xue G. (2011). PACP: An efficient pseudonymous authentication-based conditional privacy protocol for VANETs. IEEE Trans. Intell. Transp. Syst..

[15] Joshi A., Gaonkar P., Bapat J. A reliable and secure approach for efficient car-to-car communication in intelligent transportation systems. Proceedings of the 2017 International Conference on Wireless Communications, Signal Processing and Networking (WiSPNET).

[16] Lu R., Lin X., Luan T.H., Liang X., Shen X. (2011). Pseudonym changing at social spots: An effective strategy for location privacy in vanets. IEEE Trans. Veh. Technol..

[17] Thenmozhi T., Somasundaram R. (2015). Pseudonyms based blind signature approach for an improved secured communication at social spots in VANETs. Wirel. Pers. Commun..

[18] Rajput U., Abbas F., Oh H. (2016). A hierarchical privacy preserving pseudonymous authentication protocol for VANET. IEEE Access.

[19] Asghar M., Doss R.R.M., Pan L. A scalable and efficient PKI based authentication protocol for VANETs. Proceedings of the 2018 28th International Telecommunication Networks and Applications Conference (ITNAC).

[20] Förster D., Kargl F., Löhr H. PUCA: A pseudonym scheme with user-controlled anonymity for vehicular ad-hoc networks (VANET). Proceedings of the 2014 IEEE Vehicular Networking Conference (VNC).

[21] Sun Y., Zhang B., Zhao B., Su X., Su J. (2015). Mix-zones optimal deployment for protecting location privacy in VANET. Peer Peer Netw. Appl..

[22] Zhang C., Ho P.H., Tapolcai J. (2011). On batch verification with group testing for vehicular communications. Wirel. Netw..

[23] Lee C.C., Lai Y.M. (2013). Toward a secure batch verification with group testing for VANET. Wirel. Netw..

[24] Jianhong Z., Min X., Liying L. (2014). On the security of a secure batch verification with group testing for VANET. Int. J. Netw. Secur..

[25] Zhong H., Han S., Cui J., Zhang J., Xu Y. (2019). Privacy-preserving authentication scheme with full aggregation in VANET. Inf. Sci..

[26] Azees M., Vijayakumar P., Deboarh L.J. (2017). EAAP: Efficient anonymous authentication with conditional privacy-preserving scheme for vehicular ad hoc networks. IEEE Trans. Intell. Transp. Syst..

[27] Zhang L., Wu Q., Domingo-Ferrer J., Qin B., Hu C. (2016). Distributed aggregate privacy-preserving authentication in VANETs. IEEE Trans. Intell. Transp. Syst..

[28] Bayat M., Barmshoory M., Pournaghi S.M., Rahimi M., Farjami Y., Aref M.R. (2020). A new and efficient authentication scheme for vehicular ad hoc networks. J. Intell. Transp. Syst..

[29] Bayat M., Pournaghi M., Rahimi M., Barmshoory M. (2020). NERA: A New and Efficient RSU based Authentication Scheme for VANETs. Wirel. Netw..

[30] Pournaghi S.M., Zahednejad B., Bayat M., Farjami Y. (2018). NECPPA: A novel and efficient conditional privacy-preserving authentication scheme for VANET. Comput. Netw..

[31] Al-Shareeda M.A., Anbar M., Manickam S., Hasbullah I.H. (2021). SE-CPPA: A Secure and Efficient Conditional Privacy-Preserving Authentication Scheme in Vehicular Ad-Hoc Networks. Sensors.

[32] He D., Zeadally S., Xu B., Huang X. (2015). An efficient identity-based conditional privacy-preserving authentication scheme for vehicular ad hoc networks. IEEE Trans. Inf. Forensics Secur..

[33] Asaar M.R., Salmasizadeh M., Susilo W., Majidi A. (2018). A secure and efficient authentication technique for vehicular ad-hoc networks. IEEE Trans. Veh. Technol..

[34] Al-Shareeda M.A., Anbar M., Manickam S., Hasbullah I.H. (2021). Towards identity-based conditional privacy-preserving authentication scheme for vehicular ad hoc networks. IEEE Access.

[35] Li J., Choo K.K.R., Zhang W., Kumari S., Rodrigues J.J., Khan M.K., Hogrefe D. (2018). EPA-CPPA: An efficient, provably-secure and anonymous conditional privacy-preserving authentication scheme for vehicular ad hoc networks. Veh. Commun..

[36] Alshudukhi J.S., Mohammed B.A., Al-Mekhlafi Z.G. (2020). Conditional Privacy-Preserving Authentication Scheme Without Using Point Multiplication Operations Based on Elliptic Curve Cryptography (ECC). IEEE Access.

[37] Alazzawi M., Lu H., Yassin A., Chen K. (2019). Efficient Conditional Anonymity with Message Integrity and Authentication in a Vehicular Ad hoc Network. IEEE Access.

[38] Zhang J., Cui J., Zhong H., Chen Z., Liu L. (2019). PA-CRT: Chinese remainder theorem based conditional privacy-preserving authentication scheme in vehicular ad-hoc networks. IEEE Trans. Depend. Secur. Comput..

[39] Alshudukhi J.S., Al-Mekhlafi Z.G., Mohammed B.A. (2021). A Lightweight Authentication with Privacy-Preserving Scheme for Vehicular Ad Hoc Networks Based on Elliptic Curve Cryptography. IEEE Access.

[40] Cui J., Wang Y., Zhang J., Xu Y., Zhong H. (2020). Full Session Key Agreement Scheme Based on Chaotic Map in Vehicular Ad hoc Networks. IEEE Trans. Veh. Technol..

[41] Zhang J., Zhong H., Cui J., Tian M., Xu Y., Liu L. (2020). Edge computing-based privacy-preserving authentication framework and protocol for 5G-enabled vehicular networks. IEEE Trans. Veh. Technol..

[42] Cui J., Chen J., Zhong H., Zhang J., Liu L. (2020). Reliable and Efficient Content Sharing for 5G-Enabled Vehicular Networks. IEEE Trans. Intell. Transp. Syst..

[43] Cui J., Zhang X., Zhong H., Ying Z., Liu L. (2019). RSMA: Reputation system-based lightweight message authentication framework and protocol for 5G-enabled vehicular networks. IEEE Internet Things J..

[44] Al-Shareeda M.A., Anbar M., Manickam S., Hasbullah I.H. (2022). Password-Guessing Attack-Aware Authentication Scheme Based on Chinese Remainder Theorem for 5G-Enabled Vehicular Networks. Appl. Sci..

